# Life-Cycle Assessment of Laboratory-Scale Extrusion of Virgin and Recycled Polyolefins: Influence of Operating Duration on Life-Cycle Inventory Development

**DOI:** 10.3390/polym18172110

**Published:** 2026-08-31

**Authors:** Junaid Saleem, Zubair Khalid Baig Moghal, Gordon Mckay

**Affiliations:** Division of Sustainable Development, College of Science and Engineering, Hamad Bin Khalifa University, Qatar Foundation, Doha P.O. Box 5825, Qatar

**Keywords:** life-cycle assessment, life-cycle inventory, twin-screw extrusion, specific energy consumption, fixed-cycle energy, mechanical recycling, polyolefins

## Abstract

Life-cycle assessment (LCA) is widely used to evaluate the environmental performance of polymer processing and mechanical recycling systems, with its reliability fundamentally dependent on the quality and representativeness of the underlying life-cycle inventory (LCI) data. Laboratory-scale extrusion is extensively employed to generate inventory data for polymer processing, plastic waste mechanical recycling, and material development; however, the influence of operating duration on representative LCI development and the resulting environmental impact assessment has received little attention. Because startup energy represents a fixed contribution to each extrusion cycle, short-duration laboratory experiments may generate non-representative life-cycle inventories, leading to systematic overestimation of specific energy consumption (SEC) and associated environmental impacts. In this study, laboratory-scale co-rotating twin-screw extrusion of commercial virgin-grade and post-consumer high-density polyethylene (HDPE) and polypropylene (PP) was investigated to develop a runtime-dependent LCI framework. Extrusion trials at 1 and 3 h were experimentally implemented, while 8 and 24 h continuous-operation cases were developed as extrapolated runtime scenarios, which were subsequently evaluated using gate-to-gate LCA to quantify climate change (CC) and cumulative energy demand (CED) associated with extrusion electricity consumption. The measured melt flow index (MFI) ranged from 0.365 to 8.497 g 10 min^−1^, reflecting differences in material flowability and throughput. Specific energy consumption decreased from 0.931–1.282 kWh kg^−1^ for 1 h operation to 0.578–0.838 kWh kg^−1^ for 24 h operation as fixed startup energy was distributed over a larger processed mass. Consequently, gate-to-gate climate change impacts decreased from 0.483–0.664 to 0.300–0.434 kg CO_2_-eq kg^−1^, while cumulative energy demand decreased from 10.564–14.547 to 6.559–9.509 MJ kg^−1^. Startup energy accounted for 36–40% of total electricity consumption during 1 h operation but less than 3% during 24 h operation, demonstrating that laboratory operating duration can substantially influence the calculated inventory under otherwise fixed modeling conditions and consequently influence calculated environmental impacts without changes in material, equipment, or processing conditions. Sensitivity analysis showed that increasing the adopted operating power from 9 to 11 kW increased SEC by approximately 13–14% at 1 h and approximately 22% at 24 h, whereas varying fixed-cycle energy by ±20% changed SEC by approximately ±7–8% at 1 h but by less than ±1% at 24 h. The results demonstrate that representative laboratory-derived extrusion inventories depend on both fixed-energy allocation and material throughput and that explicit separation of experimental observations, modeling assumptions, and extrapolated runtime scenarios can improve the transparency and comparability of polymer-processing LCI data.

## 1. Introduction

Polyethylene (PE) and polypropylene (PP) are among the most widely used commodity polyolefin thermoplastics owing to their low cost, chemical resistance, ease of processing, and broad applicability in packaging, consumer products, pipes, films, and engineering components [1,2]. Their extensive production and consumption, however, have also made them major contributors to post-consumer plastic waste [3,4]. Mechanical recycling remains one of the most practical and widely adopted approaches for recovering these polyolefins by reprocessing waste plastics into secondary raw materials suitable for further manufacturing [5,6,7]. As efforts toward a circular plastics economy continue to expand, evaluating the environmental performance of polymer processing and recycling technologies has become increasingly important, with life-cycle assessment (LCA) serving as one of the principal tools for quantifying their environmental impacts.

Life-cycle assessment (LCA) provides a systematic framework for evaluating the environmental burdens associated with products and processes throughout their life cycle. The reliability of LCA, however, fundamentally depends on the quality and representativeness of the underlying life-cycle inventory (LCI), which compiles all material and energy inputs and emissions associated with a process [8,9]. In polymer processing and mechanical recycling, electricity consumption during extrusion frequently represents one of the dominant contributors to environmental impacts. Consequently, experimentally measured extrusion energy data are commonly used to develop life-cycle inventories, making the representativeness of these measurements essential for producing reliable and comparable LCA results.

Among polymer processing technologies, twin-screw extrusion plays a central role in mechanical recycling because it enables melting, homogenization, compounding, devolatilization, and pelletization of recycled polymers under continuous processing conditions [10]. Co-rotating twin-screw extruders are extensively employed at both laboratory and industrial scales to evaluate processability, develop recycled formulations, optimize operating conditions, and simulate industrial processing prior to scale-up [11,12,13]. Laboratory-scale extrusion is therefore widely used to generate inventory data for polymer processing and recycling research. Unlike industrial production, however, laboratory experiments are often conducted over relatively short operating durations. Under these conditions, the fixed energy required to heat the extruder from ambient temperature to the processing temperature may constitute a substantial proportion of the total electricity consumed. When this startup energy is allocated over a limited quantity of processed polymer, specific energy consumption (SEC) may be systematically overestimated, potentially leading to non-representative life-cycle inventories and biased environmental impact assessments [14,15].

Specific energy consumption is determined by the relationship between total energy input and the mass of polymer processed, and is therefore influenced by both operating duration and material throughput [14,16,17,18]. Material rheology, commonly characterized by melt flow index (MFI), strongly affects throughput because polymers with lower MFI generally exhibit higher melt viscosity and greater resistance to flow during extrusion [19,20,21]. This relationship is particularly relevant for recycled polymers, whose rheological behavior often differs from that of virgin materials owing to changes induced during previous processing and service life [5,6,7,22,23]. Consequently, differences in polymer type, material origin, flow characteristics, and operating duration collectively influence experimentally measured SEC and the life-cycle inventories derived from laboratory extrusion experiments.

Previous investigations have demonstrated that extrusion energy demand depends on operating conditions, throughput, screw configuration, thermal conditions, and polymer rheology. However, less attention has been given to how fixed non-production energy associated with equipment preparation and shutdown is allocated when short laboratory experiments are converted into LCI data [14,17,18,24,25]. Likewise, numerous LCA studies have evaluated the environmental performance of polymer processing and mechanical recycling systems using experimentally derived inventory data [26]. However, this distinction is particularly important because laboratory-derived electricity inventories may subsequently be used in environmental assessments or compared with industrial-scale data even though the relative contribution of fixed-cycle energy differs substantially with runtime. Consequently, the effect of startup energy allocation on the representativeness of laboratory-derived inventories, and the resulting climate change and cumulative energy-demand impacts, remain largely unexplored. Since laboratory extrusion experiments frequently serve as the basis for LCI development, neglecting the contribution of startup energy may systematically overestimate SEC and consequently distort the resulting environmental impacts. Establishing representative inventory data is therefore essential for improving the reliability, consistency, and comparability of LCA studies involving laboratory-scale polymer processing.

The objective of this study was therefore to develop and evaluate a runtime-dependent energy-inventory framework for laboratory-scale twin-screw extrusion of virgin and post-consumer HDPE and PP. This study distinguishes experimentally implemented processing trials from modeled energy inputs and extrapolated runtime scenarios, and separates fixed-cycle energy from runtime-dependent operating energy. Particular attention is given to the combined effects of operating duration and material throughput on SEC. A practical runtime criterion based on the fractional contribution of fixed-cycle energy is additionally proposed, and sensitivity analysis is used to evaluate uncertainty associated with the adopted operating-power and fixed-energy assumptions.

## 2. Materials and Methods

### 2.1. Materials

#### 2.1.1. Virgin and Waste Polyolefins

Commercial virgin-grade HDPE and PP obtained from QAPCO (Doha, Qatar) were used as virgin reference materials and were processed in pellet form. Post-consumer HDPE and PP were obtained from discarded consumer containers. The waste polymers were washed to remove surface contamination, subjected to forced-air drying under laboratory conditions to minimize residual surface moisture, and subsequently shredded to obtain particles suitable for feeding to the extruder.

The four materials were processed using the same laboratory extrusion system under defined material-specific processing conditions. The virgin and post-consumer materials were not selected as rheologically matched grades; instead, they represented commercially available virgin materials and realistic post-consumer feedstocks. Consequently, comparisons between virgin and waste materials are interpreted with consideration of their different rheological characteristics rather than as an isolated effect of recycling history.

#### 2.1.2. Melt Flow Index

The melt flow index (MFI) of each polymer was determined according to ASTM D1238 using a standard melt flow index tester and was used as an indicator of melt flow behavior [27]. Measurements were performed in triplicate and are reported as mean ± standard deviation.

The measured MFI values are presented in Table 1. Because MFI is inversely related to melt viscosity, it was used to qualitatively interpret differences in material flowability, and qualitative differences in processability. However, MFI was not used directly in the life-cycle inventory calculations.

The differences in MFI demonstrate that the virgin and post-consumer materials were rheologically dissimilar. In particular, the lower MFI values of the waste polymers indicate greater resistance to melt flow. However, MFI differences may arise from several factors, including molecular-weight distribution, previous thermal history, fillers, contamination, and compositional variability. These factors were not independently characterized in the present study. MFI was therefore used only for qualitative interpretation of flow behavior and was not used to calculate throughput or electricity consumption.

### 2.2. Laboratory Extrusion System

A laboratory-scale co-rotating twin-screw extruder (Brabender Lab-Compounder KETSE 20/40 D, Brabender GmbH & Co. KG, Duisburg, Germany) was used to process virgin and post-consumer waste HDPE and PP under controlled operating conditions. The extruder was equipped with twin screws of 20 mm diameter and a length-to-diameter (L/D) ratio of 40, and was driven by an 11 kW motor. A nominal screw speed of 100 rpm was adopted for the investigated processing conditions. Four independently controlled barrel heating zones enabled precise temperature control during extrusion. The nominal processing capacity of the system was 0.5–20 kg h^−1^.

HDPE was processed within a barrel temperature range of 180–220 °C, whereas PP was processed at 210–250 °C to ensure stable melt flow under laboratory conditions. These values represent the polymer-specific processing-temperature ranges and should not be interpreted as individual barrel-zone temperature profiles. For development of the runtime-dependent energy model, a representative operating power of 9 kW was adopted for all four materials. This value represents an engineering modeling input rather than a direct whole-system electrical measurement. Material-specific throughputs were adopted at 14 kg h^−1^ for virgin HDPE, 11 kg h^−1^ for waste HDPE, 16 kg h^−1^ for virgin PP, and 13 kg h^−1^ for waste PP. The measured MFI values were used only to support qualitative interpretation of rheological differences and were not used to derive these throughput inputs.

The extrusion process consisted of continuous feeding, melting, mixing, conveying, and discharge through the die, and trials were repeated three times to confirm stable processing behavior. However, electricity consumption was not independently measured using an external power analyzer; consequently, the energy results presented below are deterministic calculations based on the adopted energy-model parameters rather than replicate electrical measurements, and served as the basis for developing runtime-dependent electricity inventories used in the subsequent life-cycle assessment. These throughput values were adopted as representative scenario inputs within the nominal processing-capacity range of the extruder and should not be interpreted as direct mass-flow measurements.

### 2.3. Experimental Design

Four operating durations of 1, 3, 8, and 24 h were considered to represent progressively longer processing campaigns. The 1 h case represents a short laboratory trial, while the 3 h case represents an intermediate laboratory operating period. The 8 h case was selected as an extended or approximately working-shift-scale scenario, and the 24 h case represents an extended continuous-operation scenario. Laboratory extrusion trials were physically implemented for the 1 and 3 h operating periods and were repeated three times under the defined processing conditions. The 8 and 24 h cases were developed as extrapolated scenarios to investigate convergence of the energy inventory as fixed-cycle energy becomes progressively distributed over larger processed masses. Each operating scenario assumed a single cold start followed by continuous steady-state extrusion for the specified runtime. The end of the ramp-up period was operationally defined as the point at which the prescribed processing-temperature conditions had been reached and material feeding was initiated.

Fixed-cycle energy, representative operating power, and material-specific throughput were adopted as modeling inputs under the processing conditions described in Section 2.2. Fixed-cycle energy comprised the estimated ramp-up energy together with assigned post-operation auxiliary energy. Operating energy was calculated using the adopted operating power of 9 kW, while the processed mass for each scenario was determined from the corresponding adopted material-specific throughput and operating duration.

The modeling framework enabled comparison of total electricity consumption, processed mass, and specific energy consumption across the investigated runtime scenarios under a common set of energy-model assumptions. Within each material, the change in SEC with increasing runtime reflects the progressive dilution of fixed-cycle energy. Differences among the four materials additionally reflect the adopted material-specific throughputs and differences in material processability. Accordingly, cross-material differences are not attributed to operating duration alone. The operating conditions used to construct the runtime-dependent scenarios are summarized in Table 2.

### 2.4. Development of Runtime-Dependent Life-Cycle Inventories

Runtime-dependent life-cycle inventories were calculated from the adopted fixed-cycle energy, operating power, material-specific throughput and operating duration. The inventory development framework consisted of four sequential steps: (i) quantification of fixed-cycle energy, (ii) determination of representative steady-state running power, (iii) calculation of total electricity consumption for different operating durations, and (iv) normalization of electricity consumption to the processed polymer mass to generate inventory data on a functional basis.

Fixed-cycle energy was defined as the combined energy assigned to equipment ramp-up and post-operation activities:

(1) $$E_{\mathrm{fixed}}=E_{\mathrm{ramp-up}}+E_{\mathrm{post}}$$

where $E_{\mathrm{fixed}}$ is the fixed-cycle energy (kWh), $E_{\mathrm{ramp-up}}$ is the electricity consumption during ramp-up or startup (kWh), and $E_{\mathrm{post}}$ is the electricity consumption during post-operation activities (kWh). The baseline fixed-cycle energy was estimated as 5.1 kWh for HDPE and 5.9 kWh for PP. These values were treated as engineering modeling inputs rather than direct whole-system electrical measurements.

The total electricity consumption for each extrusion scenario was calculated as:

(2) $$E_{\mathrm{total}}=E_{\mathrm{fixed}}+P_{\mathrm{run}}t$$

where $E_{\mathrm{total}}$ is the total electricity consumption (kWh), $E_{\mathrm{fixed}}$ is the fixed-cycle energy (kWh), $P_{\mathrm{run}}$ is the adopted operating power (kW), and t is the extrusion runtime (h).

For the baseline model, the runtime-dependent relationships for the investigated polymers were:

(3) $$E_{\mathrm{total},\mathrm{HDPE}}=5.1\;\left(kWh\right)+9\;\left(kW\right)\;t\;(h)$$

(4) $$E_{\mathrm{total},\mathrm{PP}}=5.9\;\left(kWh\right)+9\;\left(kW\right)\;t\;(h)$$

where the constants (5.1 and 5.9 kWh) represent the baseline fixed-cycle energy for HDPE and PP, respectively, while 9 kW corresponds to the adopted baseline operating power.

Processed mass was calculated from the adopted throughput and runtime:

(5) m=Q . t

Total electricity consumption was subsequently normalized to the processed polymer mass to obtain the specific energy consumption (SEC):

(6) $$SEC=\frac{E_{\mathrm{total}}}{m}=\;\frac{E_{\mathrm{fixed}}}{Q\;.\;t}+\frac{P_{\mathrm{run}}}{Q}$$

where SEC is the specific energy consumption (kWh kg^−1^) and m is the mass of polymer processed (kg). The first term *[E_fixed_/(Qt)]* represents the fixed-cycle energy contribution per unit mass and decreases with increasing runtime, whereas the second term *[P_run_/Q]* represents the operating-energy contribution per unit mass under the adopted power and throughput conditions. Accordingly, runtime effects were assessed within each material, while differences among materials were interpreted in conjunction with the adopted material-specific throughputs.

The resulting runtime-dependent electricity inventories were developed separately for virgin HDPE, waste HDPE, virgin PP, and waste PP, and subsequently used as the primary life-cycle inventory input for the environmental assessment described in Section 2.5.

### 2.5. Life-Cycle Assessment

A gate-to-gate life-cycle assessment (LCA) was conducted to evaluate the environmental implications of runtime-dependent electricity consumption during laboratory-scale extrusion. The functional unit was defined as 1 kg of extruded polymer at the die outlet. The system boundary included the fixed cycle energy associated with equipment ramp-up and post-operation activities together with the operating energy associated with extrusion. A 100% net mass yield was assumed within the extrusion-stage model. Recoverable material associated with startup, shutdown, or off-spec extrusion was assumed to be returned to the feed stream; therefore, no permanent polymer loss was assigned in the baseline inventory. Permanent material losses were not independently quantified. Upstream processes, including virgin polymer production, waste collection, transportation, washing, drying, shredding, equipment manufacture, product use, and end-of-life treatment, were excluded from the assessment to isolate the influence of extrusion-stage energy on life-cycle inventory development.

Runtime-dependent electricity inventories developed in Section 2.4 were modeled in LCA for Experts (formerly GaBi) Professional 2024 using Sphera background database. Electricity consumption was modeled using the dataset “DE: Electricity from natural gas; AC, mix of direct and CHP, technology mix regarding firing and flue gas cleaning; production mix, at power plant; 1–60 kV; Power from natural gas.” A Qatar-specific natural-gas electricity dataset was not available within the database access used for this study; therefore, the German natural-gas electricity dataset was adopted as a technological proxy for the background electricity supply. Accordingly, the absolute environmental impact values should not be interpreted as representing the Qatar national electricity grid. The same electricity dataset was applied consistently to all investigated scenarios so that the influence of runtime and extrusion-energy assumptions could be evaluated on a common basis. Climate change impacts were characterized using ReCiPe 2016 Midpoint (H) and reported as kg CO_2_-eq per kg of extruded polymer. Energy demand was evaluated using the Sphera indicator “Energy (net calorific value)” and reported in MJ per kg of extruded polymer.

### 2.6. Sensitivity Analysis

A deterministic one-factor-at-a-time sensitivity analysis was performed to evaluate uncertainty associated with the principal energy-model assumptions. The baseline model used an adopted operating power of 9 kW. To assess the influence of this assumption, an additional scenario using the manufacturer-rated drive capacity of 11 kW was evaluated while maintaining fixed-cycle energy, throughput and runtime unchanged.

Sensitivity to fixed-cycle energy was evaluated independently by varying the baseline values of 5.1 kWh for HDPE and 5.9 kWh for PP by ±20%, while maintaining the adopted operating power at 9 kW and keeping all other model parameters unchanged. The sensitivity scenarios were evaluated for all four materials and operating durations of 1, 3, 8, and 24 h. Because the same background electricity dataset was retained in all scenarios, relative changes in electricity-related climate change and energy-demand results follow the corresponding changes in SEC.

## 3. Results and Discussion

### 3.1. Runtime-Dependent Electricity Consumption

The calculated electricity consumption exhibited a linear increase with operating duration for both HDPE and PP extrusion, reflecting the combined contribution of fixed-cycle energy and steady-state extrusion power. The total electricity consumption was described by Equations (3) and (4), which incorporate the baseline fixed-cycle energy inputs of 5.1 kWh for HDPE and 5.9 kWh for PP, together with a representative steady-state running power of 9 kW.

**Figure 1:**
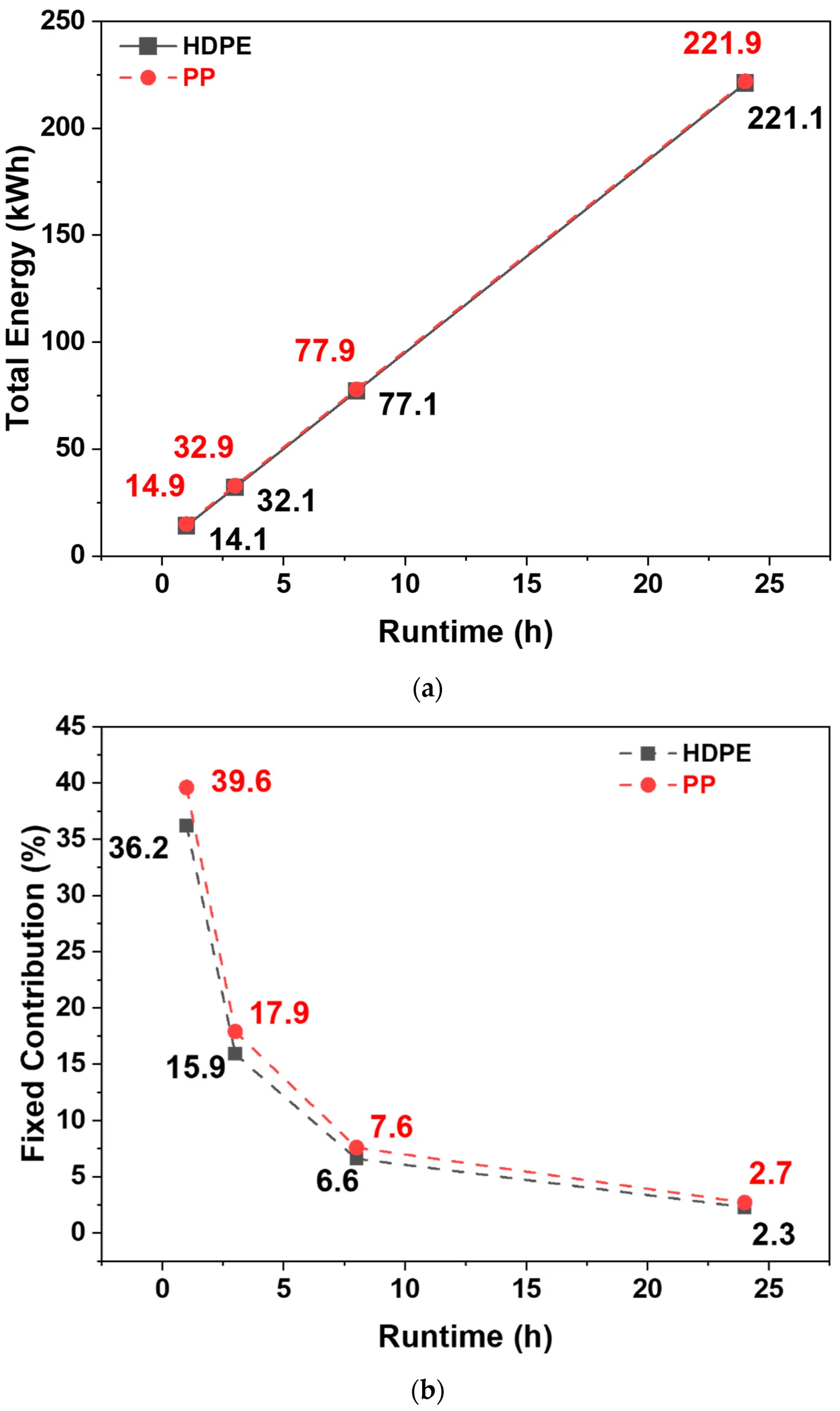
(**a**) Total electricity consumption as a function of runtime for HDPE and PP. (**b**) Contribution of fixed startup energy, including ramp-up and post-operation auxiliary energy, to total electricity consumption.

Figure 1a illustrates the total electricity consumption for the four investigated operating durations (1, 3, 8, and 24 h). Although the total electricity demand increased linearly with operating duration, the relative contribution of startup energy progressively decreased because the fixed-energy requirement was distributed over larger quantities of processed polymer. Figure 1b illustrates the corresponding decrease in the contribution of fixed-cycle energy to the total electricity consumption.

For the shortest operating duration (1 h), startup energy accounted for a substantial fraction of the total electricity demand, representing approximately 36.2% and 39.6% for HDPE and PP, respectively. As the operating duration increased to 24 h, these contributions decreased to approximately 2.3% and 2.7%, indicating that electricity consumption became increasingly dominated by steady-state extrusion.

The slightly higher startup energy observed for PP resulted from its higher processing temperature (210–250 °C) compared with HDPE (180–220 °C), requiring greater energy input during barrel heating before extrusion commenced. In contrast, the steady-state electricity demand remained essentially identical for both polymer systems because the same laboratory extruder was operated under comparable processing conditions.

These results demonstrate that laboratory-scale extrusion electricity consumption consists of two fundamentally different components: a fixed startup energy independent of operating duration and a variable steady-state energy proportional to runtime. Consequently, the influence of startup energy decreases progressively with increasing operating duration, improving the representativeness of the resulting life-cycle inventories.

### 3.2. Runtime-Dependent Specific Energy Consumption

Previous extrusion-energy studies have similarly shown that specific energy demand is strongly dependent on equipment configuration, throughput, screw geometry, processing temperature and material rheology [14,17,18,24,25]. Consequently, absolute SEC values obtained using different extrusion systems should be compared cautiously. The principal methodological contribution of the present work is therefore the explicit separation of fixed-cycle and runtime-dependent energy contributions rather than the establishment of a universal SEC benchmark.

**Figure 2:**
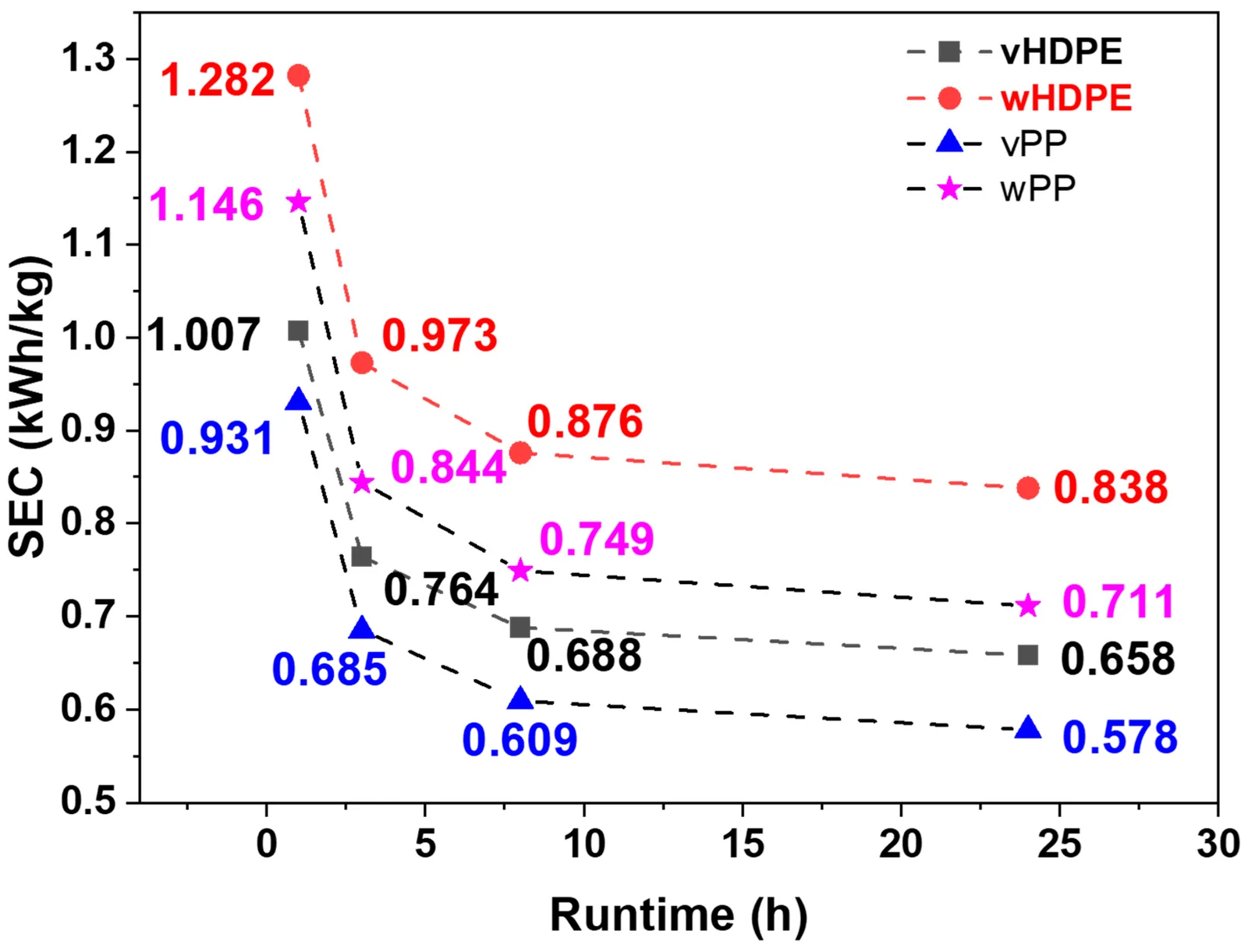
Runtime-dependent specific energy consumption (SEC) of virgin and post-consumer waste HDPE and PP during laboratory-scale twin-screw extrusion.

Specific energy consumption (SEC) was evaluated to determine how operating duration influences the electricity requirement per unit mass of processed polymer. Unlike total electricity consumption, which increased linearly with runtime, SEC decreased markedly as operating duration increased because the fixed startup energy was distributed over progressively larger quantities of processed polymer (Figure 2).

For all investigated materials, the highest SEC values were observed at the shortest operating duration (1 h), where startup energy represented a substantial proportion of the total electricity demand. As runtime increased from 1 to 24 h, SEC declined rapidly before gradually approaching an asymptotic value corresponding to steady-state extrusion. The complete runtime-dependent electricity inventory data, including startup energy, running energy, total electricity consumption, processed mass, and SEC, are summarized in Table 3. This trend demonstrates that the influence of startup energy becomes progressively less significant during extended operation.

Differences in SEC were also observed among the investigated polymers. Owing to their higher processing throughputs, virgin HDPE and virgin PP exhibited lower SEC values than their corresponding waste materials. In contrast, the lower throughputs of waste HDPE and waste PP resulted in higher electricity consumption per kilogram of processed polymer despite identical steady-state power requirements. These findings indicate that throughput plays an important role in determining SEC because greater material productivity distributes both fixed startup energy and variable operating energy over a larger processed mass.

The contributions of runtime and material throughput can be distinguished by expressing SEC as *SEC = [E_fixed_/(Qt)] + [P_run_/Q]*. The first term represents the fixed-cycle energy contribution per unit mass and decreases as runtime increases, whereas the second term represents the runtime-independent operating-energy contribution per unit mass under the adopted power and throughput conditions. Consequently, the decrease in SEC within each material with increasing runtime results from dilution of fixed-cycle energy, while differences in SEC among the four materials are additionally influenced by the adopted material-specific throughputs.

The reduction in SEC with increasing runtime has important implications for life-cycle inventory development. Laboratory experiments conducted over short operating periods systematically overestimate the electricity requirement per kilogram of processed polymer because startup energy represents a disproportionately large share of the total electricity consumption. Consequently, life-cycle inventories derived from short-duration experiments may overestimate the environmental burdens associated with polymer extrusion when used in subsequent life-cycle assessments.

The observed convergence of SEC at longer operating durations indicates that extended laboratory experiments provide more representative electricity inventories by better approximating steady-state processing conditions. Therefore, operating duration should be explicitly considered when developing laboratory-derived life-cycle inventories intended for environmental assessment or comparison with industrial-scale polymer processing.

### 3.3. Development of Representative Life-Cycle Inventories and Runtime Criterion

The experimentally determined electricity consumption data were converted into runtime-dependent life-cycle inventories by normalizing the total electricity demand to the corresponding mass of extruded polymer. Separate electricity inventories were developed for virgin HDPE, waste HDPE, virgin PP, and waste PP for each operating duration (1, 3, 8, and 24 h), thereby providing the inventory data used for the subsequent environmental assessment.

The runtime-dependent inventories directly reflected the trends observed in the specific energy consumption results presented in Section 3.2. As operating duration increased, the electricity inventory per kilogram of processed polymer decreased because the fixed startup energy was distributed over progressively larger quantities of material. Consequently, inventories derived from short-duration laboratory experiments contained higher electricity requirements than those developed from extended operating periods.

Material throughput also influenced the resulting inventories. Virgin HDPE and virgin PP exhibited lower electricity inventories than their waste counterparts owing to their higher processing throughputs, whereas waste HDPE and waste PP required greater electricity per kilogram of processed polymer despite operating under identical steady-state power conditions. These findings demonstrate that representative inventory development depends on both operating duration and material productivity.

The proposed runtime-dependent inventory framework provides a practical methodology for improving the representativeness, transparency, and comparability of laboratory-derived life-cycle inventories. Rather than relying on electricity measurements obtained from a single operating duration, the framework explicitly accounts for the influence of startup energy and enables the development of inventories that more accurately represent steady-state laboratory extrusion. These inventories subsequently served as the life-cycle inventory input for the climate change and cumulative energy-demand assessments presented in Section 3.4 and Section 3.5.

The fractional fixed-cycle contribution is expressed as:

(7) $$f_{\mathrm{fixed}}=\;\frac{E_{\mathrm{fixed}}}{E_{\mathrm{fixed}}+\;P_{\mathrm{run}}\;.\;t}$$

A representative laboratory runtime can be selected by defining a maximum acceptable contribution of fixed-cycle energy. Using 5% as a practical criterion, the baseline model gives minimum runtimes of approximately 10.8 h for HDPE and 12.5 h for PP. Thus, approximately 13 h of operation would limit the contribution of fixed-cycle energy to about 5% or less for both polymer systems under the present assumptions. This duration should not be interpreted as a universal requirement because the corresponding value depends on equipment-specific fixed energy and operating power. The transferable recommendation is therefore to report fixed-cycle energy separately and determine the required runtime according to a predefined acceptable fixed-energy contribution.

### 3.4. Climate Change Impacts

**Figure 3:**
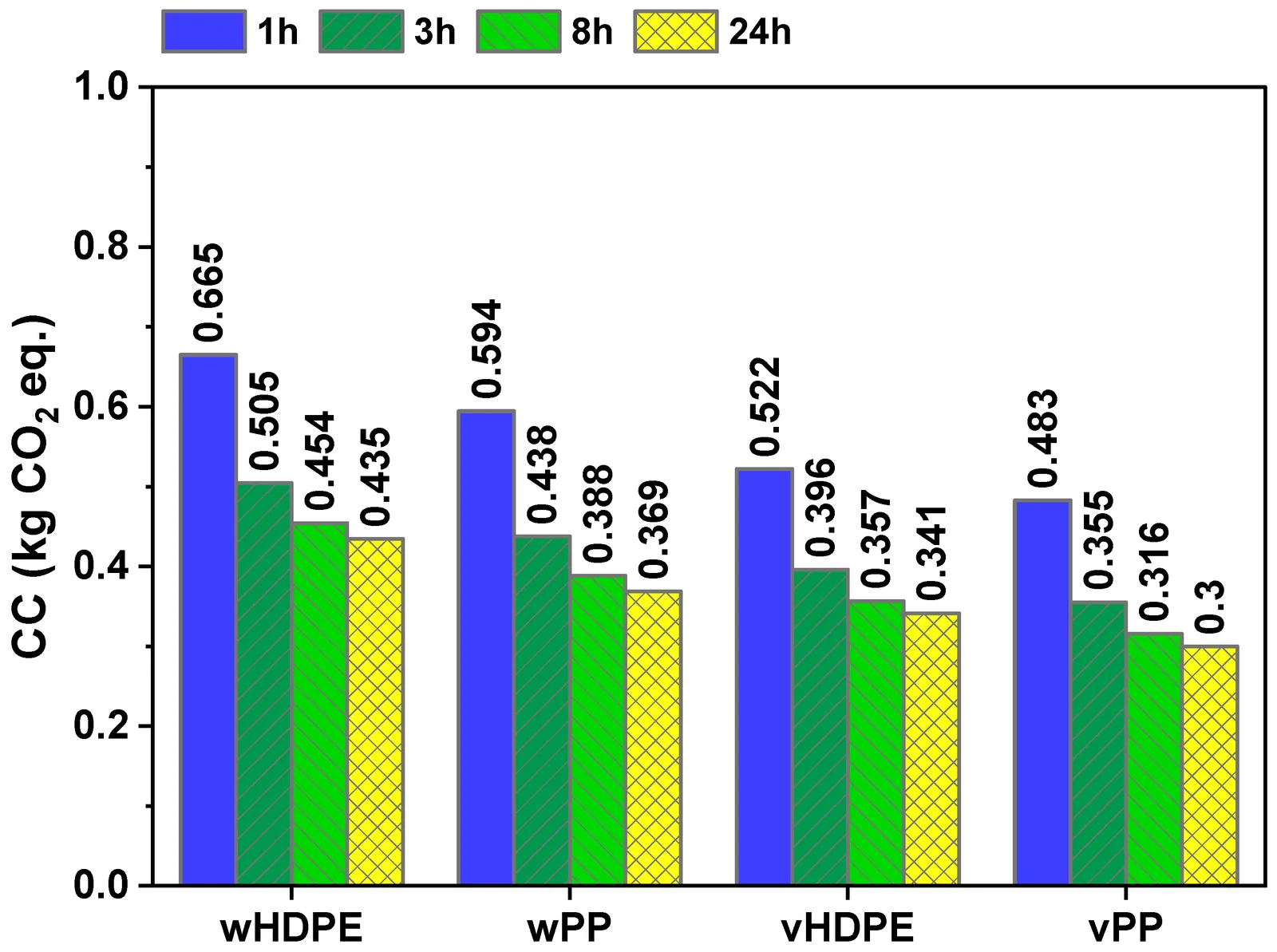
Climate change impacts of runtime-dependent LCI developed for laboratory-scale extrusion.

The runtime-dependent life-cycle inventories developed in Section 3.3 were subsequently used to evaluate the climate change (CC) impacts associated with laboratory-scale extrusion. Figure 3 presents the CC impacts for virgin HDPE, waste HDPE, virgin PP, and waste PP under the four investigated operating durations (1, 3, 8, and 24 h).

The CC impacts decreased consistently with increasing operating duration for all investigated materials, reflecting the reduction in electricity consumption per kilogram of processed polymer associated with extended extrusion operation. The highest CC impacts were observed for the 1 h operating scenario, where the fixed startup energy represented a substantial proportion of the total electricity demand. As operating duration increased, the contribution of startup energy became progressively less significant, resulting in lower greenhouse gas emissions per kilogram of extruded polymer.

Among the investigated materials, virgin HDPE and virgin PP exhibited lower CC impacts than their waste counterparts owing to their higher processing throughputs, and consequently lower electricity requirements per unit mass. In contrast, waste HDPE and waste PP generated comparatively higher emissions because the same steady-state electricity demand was distributed over smaller quantities of processed polymer.

The reduction in CC impacts became progressively smaller beyond intermediate operating durations, indicating convergence toward steady-state environmental performance. This behavior demonstrates that inventories developed from extended laboratory operation provide more representative estimates of the greenhouse gas emissions associated with continuous polymer extrusion.

These findings highlight the importance of considering operating duration when developing laboratory-derived life-cycle inventories. Environmental assessments based solely on short-duration laboratory experiments may systematically overestimate climate change impacts because of the disproportionate influence of startup energy. Incorporating runtime-dependent life-cycle inventories therefore improves the representativeness, consistency, and comparability of climate change assessments for laboratory-scale polymer processing and mechanical recycling systems.

### 3.5. Cumulative Energy Demand

**Figure 4:**
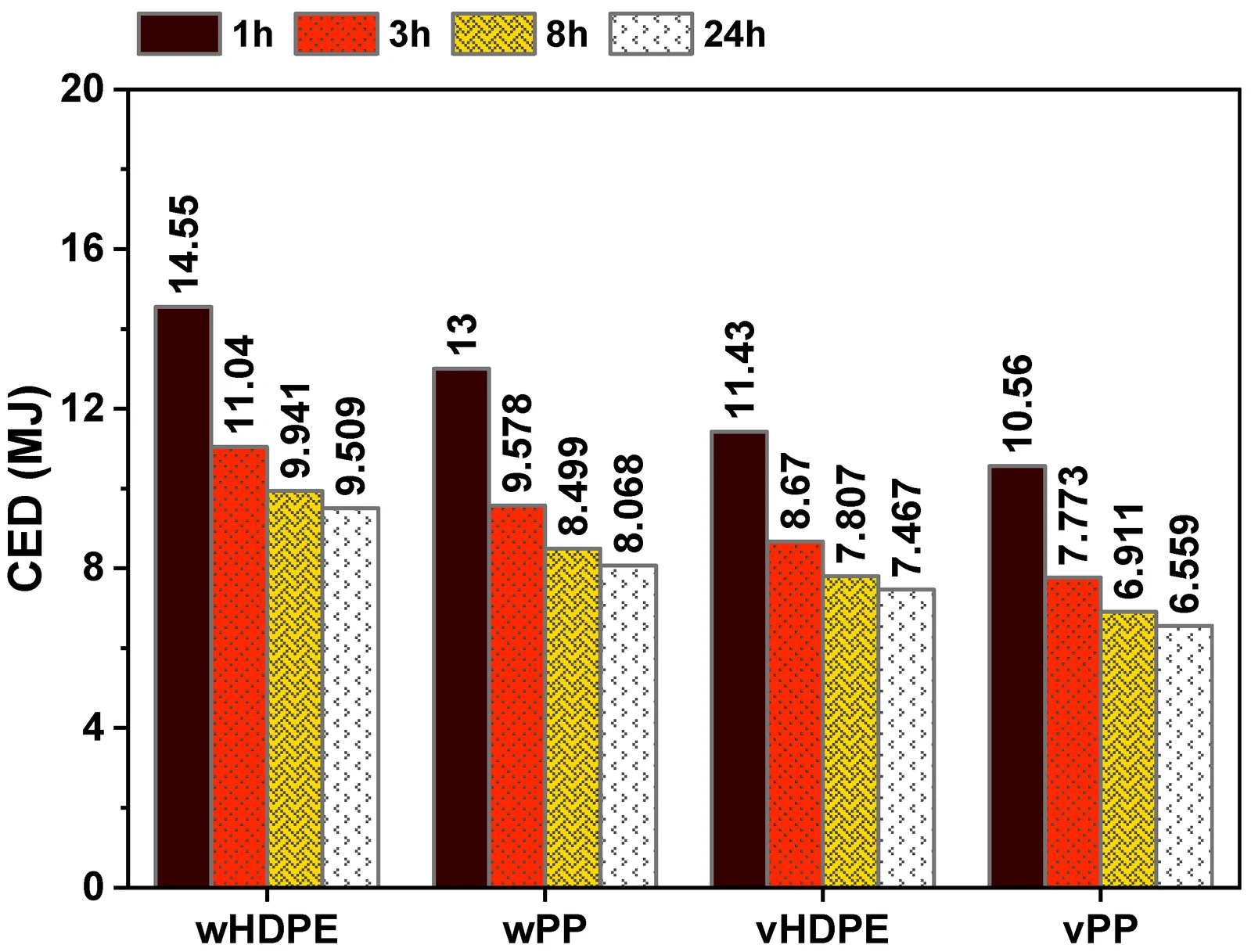
Cumulative energy demand (CED) of runtime-dependent LCI developed for extrusion.

The runtime-dependent life-cycle inventories were further evaluated in terms of cumulative energy demand (CED) to quantify the total primary energy associated with electricity consumption during laboratory-scale extrusion. Figure 4 presents the CED values for virgin HDPE, waste HDPE, virgin PP, and waste PP under the four investigated operating durations.

The CED results exhibited trends similar to those observed for climate change impacts, with progressively lower primary energy requirements as operating duration increased. The highest CED values were obtained for the 1 h operating scenario, reflecting the substantial contribution of fixed startup energy to the total electricity consumption. As operating duration increased, the influence of startup energy diminished, resulting in lower primary energy demand per kilogram of processed polymer.

Among the investigated materials, virgin HDPE and virgin PP exhibited lower CED values than their waste counterparts owing to their higher processing throughputs and correspondingly lower electricity requirements per unit mass. In contrast, waste HDPE and waste PP required greater primary energy per kilogram of processed polymer because the same steady-state electricity demand was distributed over smaller processed masses.

The reduction in CED became progressively smaller at longer operating durations, indicating convergence toward steady-state energy performance. This behavior demonstrates that inventories developed from extended laboratory operation provide more representative estimates of the primary energy demand associated with continuous polymer extrusion.

These results further demonstrate that operating duration is an important consideration when developing laboratory-derived life-cycle inventories. Inventories generated from short-duration experiments may systematically overestimate cumulative energy demand because of the disproportionate contribution of startup energy. Incorporating runtime-dependent life-cycle inventories therefore improves the representativeness, consistency, and comparability of energy-based environmental assessments for laboratory-scale polymer processing and mechanical recycling systems.

### 3.6. Sensitivity Analysis

**Figure 5:**
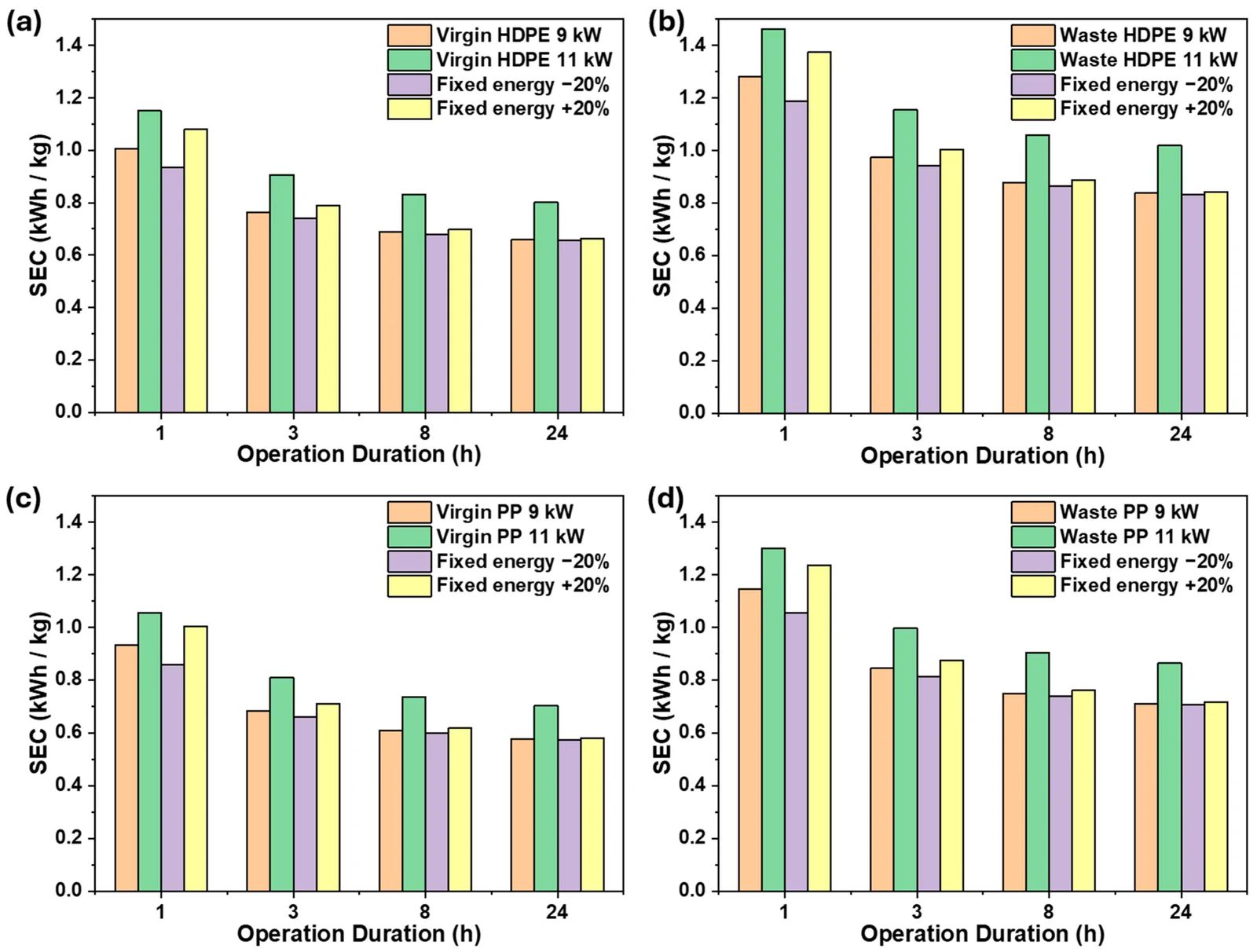
Sensitivity of specific energy consumption (SEC) to the adopted operating-power and fixed-cycle-energy assumptions for (**a**) virgin HDPE, (**b**) waste HDPE, (**c**) virgin PP, and (**d**) waste PP at operating durations of 1, 3, 8, and 24 h. The baseline scenario uses an adopted operating power of 9 kW and fixed-cycle energies of 5.1 kWh for HDPE and 5.9 kWh for PP. Sensitivity scenarios include an operating power of 11 kW and ±20% variation in the baseline fixed-cycle energy. All other model parameters were held constant.

Figure 5 presents the sensitivity of SEC to changes in the adopted operating-power and fixed-cycle-energy assumptions. Increasing the operating-power assumption from the baseline value of 9 kW to 11 kW increased SEC for all four materials and all operating durations. The relative influence of operating power increased with runtime, with SEC increasing by approximately 13–14% at 1 h and approximately 22% at 24 h. This behavior reflects the increasing dominance of runtime-dependent operating energy as the contribution of fixed-cycle energy becomes progressively diluted.

In contrast, the influence of fixed-cycle-energy uncertainty was greatest during short-duration operation. A ±20% variation in fixed-cycle energy changed SEC by approximately ±7–8% at 1 h and approximately ±3–4% at 3 h. The effect decreased to approximately ±1–2% at 8 h and to less than ±1% at 24 h. Thus, uncertainty in the fixed-cycle-energy estimate becomes progressively less important as operating duration increases.

The sensitivity analysis therefore identifies two different uncertainty regimes. Accurate estimation of fixed-cycle energy is particularly important when laboratory experiments are conducted over short durations, whereas the adopted operating-power value becomes comparatively more influential during extended operation. Importantly, all sensitivity scenarios retained the same overall runtime-dependent behavior: SEC decreased as fixed-cycle energy was distributed over increasing processed mass.

Because the background electricity dataset was unchanged across the sensitivity scenarios, the relative changes in electricity-related climate change and energy-demand results follow the corresponding relative changes in SEC.

### 3.7. Applicability and Limitations

The numerical results obtained in this study are specific to the laboratory-scale extrusion system and modeling assumptions employed and should not be interpreted as direct industrial-scale benchmarks. Industrial extruders differ substantially from laboratory systems in throughput, motor loading, surface-to-volume ratio, thermal losses, barrel insulation, auxiliary equipment, process control, and continuous operating practice. Consequently, direct numerical scale-up of the reported SEC values is not appropriate. The transferable aspect of the proposed framework is the separation of fixed-cycle energy from runtime-dependent operating energy and the normalization of these contributions to processed mass.

Whole-system electrical demand was not directly measured at the equipment mains, and the individual contributions of the extruder drive, barrel heaters, cooling equipment, and control auxiliaries were therefore not resolved experimentally. The adopted 9 kW operating-power and fixed-cycle energy values should consequently be interpreted as engineering modeling inputs. In addition, the thermal insulation characteristics of the extruder barrel were not independently characterized, and heat losses associated with the barrel were not quantified separately.

The 8 and 24 h cases were extrapolated continuous-operation scenarios rather than experimentally conducted long-duration trials. Consequently, possible long-duration changes in polymer molecular weight, rheology, contamination accumulation, torque, throughput, or equipment performance were not represented in the model.

The exact commercial grade designation and PP subtype were not retained in the archived experimental record, which limits direct comparison with grade-specific industrial processing data. Permanent polymer losses were not independently quantified; the baseline model assumed 100% net extrusion-stage mass yield, with recoverable startup, shutdown, or off-spec material assumed to be returned to the feed stream. In addition, the German natural-gas electricity dataset was used as a technological proxy because a Qatar-specific dataset was unavailable within the database access used, and the resulting environmental impacts should therefore not be interpreted as Qatar-specific electricity impacts.

Application of the framework to polymers with substantially different processing temperatures and rheological behavior, including PET, PS and PA, requires polymer-specific validation.

## 4. Conclusions

This study investigated the influence of operating duration on life-cycle inventory development during laboratory-scale twin-screw extrusion of virgin and post-consumer HDPE and PP. Under the baseline modeling assumptions, total electricity consumption increased from 14.1–14.9 kWh for 1 h operation to 221.1–221.9 kWh for 24 h operation, while SEC decreased from 0.931–1.282 kWh kg^−1^ to 0.578–0.838 kWh kg^−1^ because fixed-cycle energy was distributed over increasing processed mass. Baseline fixed-cycle energy inputs of 5.1 kWh for HDPE and 5.9 kWh for PP were used as engineering modeling parameters rather than direct whole-system electrical measurements.

Using a maximum fixed-cycle contribution of 5% as a practical criterion resulted in minimum runtimes of approximately 10.8 h for HDPE and 12.5 h for PP, suggesting that approximately 13 h of operation would limit the fixed-cycle contribution to about 5% or less under the present model assumptions. Material throughput also significantly influenced the resulting runtime-dependent life-cycle inventories despite a common steady-state running power of 9 kW for all four materials. Virgin PP exhibited the lowest SEC owing to its higher throughput, whereas waste HDPE showed the highest SEC because lower material productivity distributed the fixed and variable electricity demand over a smaller processed mass.

Sensitivity analysis showed that uncertainty in fixed-cycle energy had its greatest influence during short-duration operation, whereas uncertainty in the adopted operating power became comparatively more important at extended runtimes. Nevertheless, the principal trend of decreasing SEC with increasing runtime remained unchanged across the investigated sensitivity scenarios.

The gate-to-gate climate change impacts associated with extrusion electricity ranged from 0.300 to 0.664 kg CO_2_-eq kg^−1^, while energy demand evaluated using the Sphera “Energy (net calorific value)” indicator ranged from 6.559 to 14.547 MJ kg^−1^. Extending the runtime from 1 to 24 h reduced both indicators by approximately 35–38% under otherwise fixed modeling assumptions.

Overall, the results demonstrate that laboratory operating duration is a critical determinant of representative life-cycle inventory development. By explicitly accounting for startup energy and runtime-dependent electricity consumption, the proposed framework improves the representativeness, consistency, and comparability of laboratory-derived extrusion inventories. The proposed methodology provides a practical basis for future life-cycle assessment studies involving laboratory-scale polymer processing and mechanical recycling systems.

## Figures and Tables

**Table 1:** Melt flow index of virgin and waste polyolefins.

Material	MFI (g/10 min)
Virgin HDPE	0.7475 ± 0.0118
Virgin PP	8.4967 ± 0.1297
Waste HDPE	0.3653 ± 0.0045
Waste PP	2.0443 ± 0.0422

**Table 2:** Operating conditions used during laboratory-scale extrusion.

Material	Processing Temperature (°C)	Nominal Screw Speed (rpm)	Adopted Throughput (kg h^−1^)	Adopted Operating Power (kW)
Virgin HDPE	180–220	100	14	9
Waste HDPE	180–220	100	11	9
Virgin PP	210–250	100	16	9
Waste PP	210–250	100	13	9

**Table 3:** Runtime effect on energy consumption and SEC for virgin and waste polyolefins.

Material	Runtime (h)	Ramp-Up Energy (kWh)	Running Energy (kWh)	Post-Operation Auxiliary Energy (kWh)	Total Energy (kWh)	Output (kg)	SEC (kWh kg^−1^)
Virgin HDPE	1	4.8	9.0	0.3	14.1	14	1.007
Virgin HDPE	3	4.8	27.0	0.3	32.1	42	0.764
Virgin HDPE	8	4.8	72.0	0.3	77.1	112	0.688
Virgin HDPE	24	4.8	216.0	0.3	221.1	336	0.658
Waste HDPE	1	4.8	9.0	0.3	14.1	11	1.282
Waste HDPE	3	4.8	27.0	0.3	32.1	33	0.973
Waste HDPE	8	4.8	72.0	0.3	77.1	88	0.876
Waste HDPE	24	4.8	216.0	0.3	221.1	264	0.838
Virgin PP	1	5.5	9.0	0.4	14.9	16	0.931
Virgin PP	3	5.5	27.0	0.4	32.9	48	0.685
Virgin PP	8	5.5	72.0	0.4	77.9	128	0.609
Virgin PP	24	5.5	216.0	0.4	221.9	384	0.578
Waste PP	1	5.5	9.0	0.4	14.9	13	1.146
Waste PP	3	5.5	27.0	0.4	32.9	39	0.844
Waste PP	8	5.5	72.0	0.4	77.9	104	0.749
Waste PP	24	5.5	216.0	0.4	221.9	312	0.711

## Data Availability

The original contributions presented in the study are included in the article; further inquiries can be directed to the corresponding authors.
